# To Estimate the Dampness of a House

**Published:** 1896-03

**Authors:** 


### To Estimate the Dampness of a House.

Physicians are sometimes requested to estimate the relative dampness of an apartment or room. This is not always easy by simple inspection, as a room may be damp although saltpeter does not grow on its walls or mould in its corners. The following is an exact means of appreciation, and one that is within every one's scope. In the room in question a kilogramme of fresh lime should be placed after hermetically closing doors and windows. In twenty-four hours it should be weighed, and if the kilogramme has absorbed more than ten grammes of water (that is, more than one percent), the room should be considered damp and classed as unhealthy.

The question of the dampness of dwellings is a frequent cause of dispute between landlord and tenant, naturally solved in the affirmative by the latter, and in the negative by the former. The question can be settled in the future by the test of the hydratation of lime, of which I have just spoken, and which will give irrefutable proof of the validity of such complaint.—*European Edition N. Y. Herald.*

